# First muography of Stromboli volcano

**DOI:** 10.1038/s41598-019-43131-8

**Published:** 2019-04-30

**Authors:** Valeri Tioukov, Andrey Alexandrov, Cristiano Bozza, Lucia Consiglio, Nicola D’Ambrosio, Giovanni De Lellis, Chiara De Sio, Flora Giudicepietro, Giovanni Macedonio, Seigo Miyamoto, Ryuichi Nishiyama, Massimo Orazi, Rosario Peluso, Andrey Sheshukov, Chiara Sirignano, Simona Maria Stellacci, Paolo Strolin, Hiroyuki K. M. Tanaka

**Affiliations:** 1 Istituto Nazionale di Fisica Nucleare, Sezione di Napoli, Napoli, Italy; 2 Dipartimento di Fisica, Università degli Studi di Salerno, Fisciano, Italy; 3 Istituto Nazionale di Fisica Nucleare, LNGS, L’Aquila, Italy; 4 Dipartimento di Fisica E.Pancini, Università degli Studi di Napoli Federico II, Napoli, Italy; 5 Istituto Nazionale di Geofisica e Vulcanologia, Osservatorio Vesuviano, Napoli, Italy; 6 Earthquake Research Institute, The University of Tokyo, Tokyo, Japan; 7 Joint Institute for Nuclear Research, Dubna, Russia; 8 Dipartimento di Fisica e Astronomia, Università degli Studi di Padova, Padova, Italy

**Keywords:** Geophysics, Volcanology, Experimental particle physics

## Abstract

Muography consists in observing the differential absorption of muons – elementary particles produced through cosmic-ray interactions in the Earth atmosphere – going through the volcano and can attain a spatial resolution of tens of meters. We present here the first experiment of nuclear emulsion muography at the Stromboli volcano. Muons have been recorded during a period of five months by a detector of 0.96 m^2^ area. The emulsion films were prepared at the Gran Sasso underground laboratory and were analyzed at Napoli, Salerno and Tokyo scanning laboratories. Our results highlight a significant low-density zone at the summit of the volcano with density contrast of 30–40% with respect to bedrock. The structural setting of this part of the volcanic edifice controls the eruptive dynamics and the stability of the “Sciara del Fuoco” slope, which is affected by recurrent tsunamigenic landslides. Periodical imaging of the summit of the Stromboli volcano such as that provided by muography can become a useful method for studying the evolution of the internal structure of the volcanic edifice.

## Introduction

Muography was first applied in 1970 by the Particle Physics Nobelist Luis Alvarez and collaborators to the search for hidden chambers in the Chephren pyramid^1^. The breakthrough in volcano muography occurred with the upper part of the edifice of the Asama volcano in Japan^2^. Muon absorption radiography, or *muography*, is conceptually similar to standard X-ray radiography. The role of X-rays is played by *muons*, penetrating particles similar to electrons but with a mass about 200 times higher that are suitable to image the internal structure of large objects such as volcano edifices. A natural flux of muons from the sky is available at the Earth surface. Stable particles (mostly protons and He nuclei) travel galactic or intergalactic distances and impinge on the atoms of the Earth atmosphere, producing showers of particles. Mesons in the showers decay at high altitudes to muons that reach the ground. At sea level, the energy spectrum of muons extends to 10^3^ GeV and beyond. The high-energy component can travel rock thickness comparable with the size of volcano edifices. The muon flux after crossing a volcanic edifice carries directional information about absorption in the volcano itself. It can thus provide a map – in angular coordinates – of the average rock density encountered inside the volcano, the length of the path being known from Digital Elevation Models (DEM). Such density map can give indications for internal structures and eruption or magma dynamics, as shown in^3^ and^4^. Combined analyses can also be carried together with the data gathered by conventional geophysical techniques, as reported in^5^ and in^6^ who jointly inverted gravimetric data together with 3D muon data from three simultaneous telescope acquisitions. Other muography experiments to study the internal structure of volcanoes have been carried out in Japan^4,7^, France^8–11^ and Italy^12–14^. The thicker the volcano edifice, the stronger the muon flux attenuation, the longer the exposure and the larger the detector area needed to integrate enough statistics in the detector. Data-taking campaigns have typically been performed for volcano domes with a thickness of the order of 0.5–1 km, using detectors of 1 m^2^ transverse area and with data taking periods of the order of a few months. Here we present the first muography of Stromboli volcano, which is the result of an experiment we conducted between 2011 and 2012. Stromboli is a large strato-volcano of the Aeolian archipelago (Italy), characterized by the emission of huge amounts of gas^15–18^ and by typical Strombolian explosive activity, lasting for several centuries^19–22^. The top of the volcano is about 920 m above the sea level and the submarine portion extends down to about 1000 m depth into the Tyrrhenian sea.

**Figure 1:**
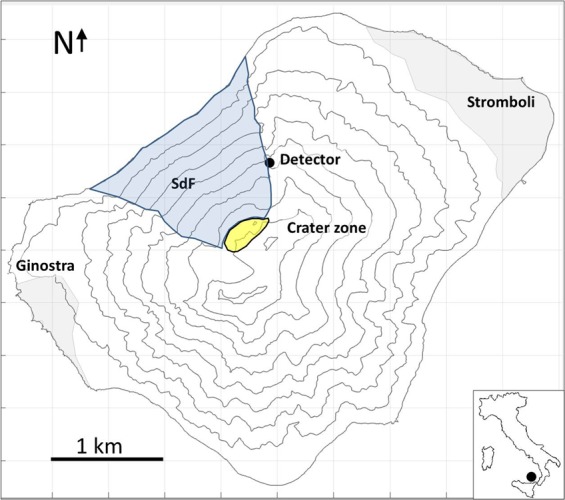
Stromboli island. Stromboli and Ginostra are the two villages that are located on the north-east and south-west coast respectively. SdF indicates the north-western side of the island called “Sciara del Fuoco”. The black spot marks the detector installation site (Le Roccette) and the yellow area indicates the crater region (graben-like collapsed zone).

Stromboli volcano shows a long volcanic history^23,24^, characterized by effusive and explosive eruptions and by repeated collapse episodes^25^. The Sciara del Fuoco, on the NW side (Fig. 1), is the result of these collapses^26^. This structure controls the present morpho-structural setting of the island^27^. The Sciara del Fuoco slope consists mainly of coarse loose pyroclastic material produced by the persistent Strombolian explosive activity intercalated with lava flows, which formed up during effusive phases. These materials fill the depression created by the last sector collapse, which occurred about 5 ka ago^25^.

A mechanism based on destructive episodes, due to the collapse of the volcano flank, and the subsequent reconstruction through eruptive activity, is typical of the morphological evolution of Stromboli island, as demonstrated also by bathymetric evidence, showing large debris avalanche deposits due to repeated large-scale lateral collapses^28,29^.

The crater region of the volcano is characterized by several eruptive vents aligned along the direction of elongation of the volcanic edifice, which often form small scoria cones. The persistent low energy explosive activity of these vents provides loose pyroclastic material that covers the steep slope of Sciara del Fuoco.

The current activity of Stromboli is characterized by some hundreds of moderate explosions per day. Major explosions, which launch scoria up to hundreds of meters from the craters, and paroxysmal explosions, which produce large ballistic blocks and can cause small pyroclastic currents, sometimes take place^30^. The volcanological context of Stromboli highlights the structural fragility of the summit crater area and of the Sciara del Fuoco slope (Fig. 1), with a resulting potential hazard induced by the dynamics of the upper part of the edifice. We have thus performed a muography of the internal structure of the summit crater area, using a detector of 0.96 m^2^ area that took data for a timespan of about five months. The detector was based on the so-called nuclear emulsion technique^31^, briefly described below. We have used emulsion films^32^ designed for the Oscillation Project with Emulsion-tRacking Apparatus (OPERA) experiment^33,34^. Below we present the results of our experiment and we describe the methodological details.

## Results

### Emulsion exposure

Our experiment was focused on the study of the Stromboli crater region therefore the choice of the exposure site was optimized for the observation of this area, which is around 750 m a.s.l. Following a detailed simulation of the muon flux (as specified further on), the suitable range of altitudes was found to be between 600 and 650 m a.s.l. After a survey of the field, a site at 640 m a.s.l. (referred to as Le Roccette) was chosen.

**Figure 2:**
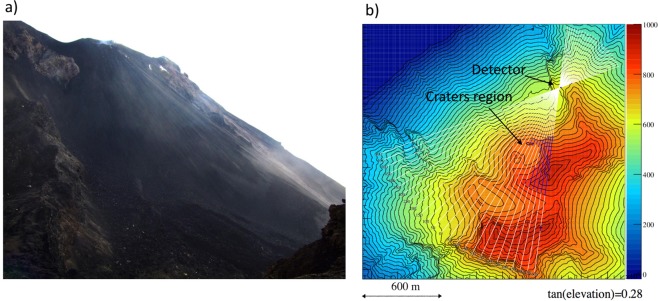
(**a**) The upper part of the Sciara del Fuoco and the crater region seen from the detector location. (**b**) Detector location and crater in a map from the Digital Elevation Model, with muon trajectories for a tangent of the elevation angle equal to 0.28 and azimuthal angle acceptance of ±0.6 rad. The color scale indicates the elevation in meters.

On the Fig. 2a is the photo of upper part of the Sciara del Fuoco and the crater region taken from the detector position. Figure 2b shows the detector location and the crater region in a map from the Digital Elevation Model (DEM), with altitudes given in color scale and contours at constant altitude given with a 10 m pitch. The straight lines indicate muon paths in the case where the tangent of elevation angle *θ*_*y*_ is 0.28, roughly corresponding to the crater region. White or blue segments mean that the path is in the air or in the rock, respectively. The horizontal angle *θ*_*x*_ is defined as the azimuthal angle referred to the pointing direction of the detector, perpendicular to the emulsion films. The detector was pre-assembled at the Civil Protection site in Stromboli and lifted up to the exposure site by helicopter.

The detector planes were placed in the vertical direction by means of standard techniques. A preliminary estimate of the azimuthal angle of the detector was done using a magnetic compass. Precision measurements were performed by a GPS system. The coordinates of two reference points at the extremities of the detector were measured with an accuracy of 5 mm. Three fixed reference points were installed near the detector to allow additional GPS measurements after the detector disassembly.

The emulsion films were hermetically sealed one by one inside laminated film envelopes in the emulsion facility at the Gran Sasso underground laboratory and transported to Stromboli. Such packaging protects emulsions from brightening, ambient humidity variations and dust contamination. They were inserted into the modules in situ, to avoid the accumulation of aligned base-tracks from cosmic-ray showers, not related to the exposure. The emulsion films were mounted in the modules inside a tent, to protect them from Sun heating and occasional volcanic dust or ash.

The exposure started on October 22, 2011 and ended on March 24, 2012 for a total duration of about five months, mainly in wintertime. The emulsion temperature was monitored all along the exposure by a data-logger mounted on the detector. The recorded temperature was inside the allowed range (with the mean value near 10 °C and maximum below 25 °C full period).

At the end of the exposure, emulsions films were extracted and packed in a different sequence, to prevent recording spurious tracks interconnected among emulsion films. The films were developed in the OPERA facility at Gran Sasso underground laboratory (LNGS, Italy), within one week from their extraction. No damage due to excessive humidity, thermal or mechanical stresses was reported just after the extraction or after the chemical development. The emulsion films corresponding to the eight detector modules were distributed among the Napoli, Salerno and Tokyo scanning laboratories. For the exposure reported here, ten-year-old emulsion films produced for the OPERA experiment were used. Nuclear emulsion suffers aging effects leading to a worsening of its sensitivity and to an increase of background grains, in particular of those thermally generated called *fog*. The resulting degradation of the track reconstruction efficiency was found to be especially significant in the emulsion films of one of the detector modules, so that they were discarded. The total analyzed emulsion area was 0.73 m^2^.

### The detector performance

#### Angular acceptance

Emulsions record tracks passed through with any angle, and all of them can be acquired by the scanning system we used^35,36^. Nevertheless, angles above *tg*(*θ*) = 0.6 are not useful for this muography since the expected statistics is limited by the geometrical acceptance decreasing as the *cos*(*θ*). We acquired tracks in a range up to 0.6 radian with respect to the telescope axis, sufficient to cover the target area.

#### Angular resolution

**Figure 3:**
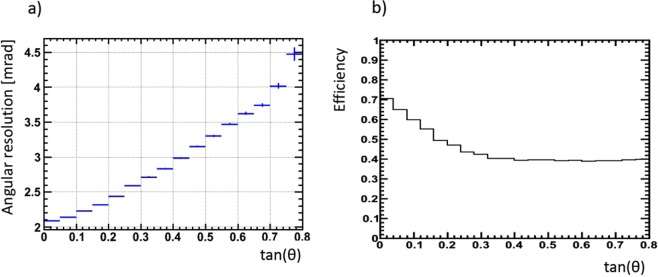
(**a**) Angular resolution of the tracking system as a function of track inclination; (**b**) Single plate basetrack efficiency.

We estimated angular resolution calculating the difference between basetracks and a fitted track angles divided by $\sqrt{3}$. Only tracks with 3 and 4 basetracks were taken into account here. This conservative way of estimation includes all contributions to the accuracy coming from scanning system and emulsion geometry, getting rid of possible distortions and alignment effects. Planarity and mechanical alignment of different modules composed the detector are not included in this estimation, but it’s expected to be less than 1 mrad by mechanical tolerances. So in the target region (with *tg*(*θ*) up to 0.4) the detector angular resolution is well below 3 mrad (Fig. 3a).

#### Momentum threshold and background rejection

**Figure 4:**
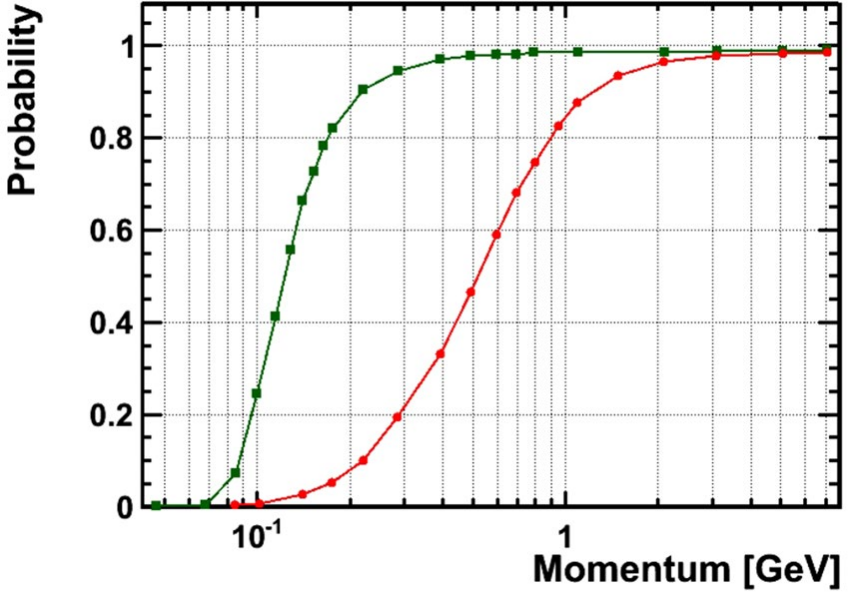
Track reconstruction probability as a function of muon momentum for two simulated configurations: 4 emulsions without absorber (green line) and with 5 mm absorber (red line). In our detector (red line) all muons below 0.1 GeV are rejected, 50% of muons at 0.5 GeV are rejected, most of the muons above 1 GeV are reconstructed.

To estimate the low-momentum rejection threshold we simulated our detector using the FLUKA^37^ package. Muons with momentum ranging from 0.5 to 10 GeV/c were traced by the simulator through the detector including all the physical effects such as multiple scattering, energy loss, interactions and were producing hits (microtracks) inside the sensitive emulsion layers. These hits were sent through the reconstruction software for tracking. Finally we compared tracks reconstructed with those simulated to measure the track reconstruction efficiency as a function of their momentum. Low momentum tracks scatter in the iron absorber by angles exceeding the tracking acceptance (15 mrad at low angles) so they don’t form a straight tracks, this is the reason why low momentum tracks are rejected. Two different geometries were simulated: four films without absorber and four films with 5 mm-thick stainless steel plate in between the films (this work) (Fig. 4).

One more type of background is the combinatorial one due to fake tracks formed by random coincidence of segments. To estimate the probability of this effect we perform a “misalignment” procedure where all 4 plates of the module are artificially shifted by 500 microns (more then 10 *σ* where *σ* is the plate-to-plate matching accuracy) in XY plane from the correct position in different directions thus excluding the possibility for real muon tracks to be matched. In this case all tracks obtained by the reconstruction are fake ones. We got the fake-to-real tracks ratio as 0.0006, where less than 1% of the fake tracks are located inside the angular region covered by the volcano, therefore the combinatorial background in this analysis is completely negligible.

#### Tracking efficiency

**Figure 5:**
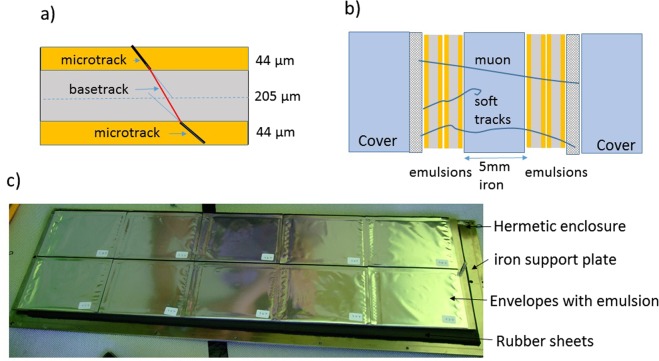
Structure of one detector module: (**a**) OPERA emulsion film cross-section and track reconstruction procedure; (**b**) cross-section of an emulsion stack where two couples of films are positioned on both sides of a 5 mm thick stainless steel plate; (**c**) Photo of the insides of one module with ten emulsion stacks positioned.

The mean efficiency of a single emulsion plate was about 50% due to aging of the 10-years old OPERA emulsions used in our installation (Fig. 3b). We applied data driven efficiency correction using the reconstructed tracks. High momentum muon tracks should be recognized in all 4 plates of two emulsion doublets separated by 5 mm of iron as shown in Fig. 5, leaving 4 base-tracks. On the contrary low momentum background including electrons and other soft showers components cannot be reconstructed as a straight track in both doublets. To reject such physical background we consider, for this muography, tracks with 3 (*N*_3_) and 4 (*N*_4_) base-tracks only. Taking into account that in this sample emulsion inefficiency is the main reason to miss the basetrack and assuming a binomial distribution for the detected base-tracks, we have estimated the number of passing-through tracks (*N*_0_) using *N*_3_ and *N*_4_. Single plate efficiency *e* is derived from *N*_4_ = *e*^4^*N*_0_ and *N*_3_ = 4*e*^3^(1 − *e*)*N*_0_ as *e* = 4*N*_4_/(*N*_3_ + 4*N*_4_). The original tracks count *N*_0_ could be derived from the above equations as *N*_0_ = *N*_4_(1 + *N*_3_/(4*N*_4_))^4^. This value was estimated for each angular bin in the acceptance range and it was used as a the measured number of muons for the analysis. This procedure for the efficiency correction was verified as following: muons generated in momentum range from 1 to 100 GeV and with wide angular spectrum were traced through the detector using the FLUKA package. The inefficiency was simulated by removing randomly hits (basetracks) from the sample according to the probability value. Afterward, the track reconstruction procedure was applied using the same acceptances as for real data, followed by the efficiency correction procedure. We observe that in the relevant efficiency range the number of tracks *N*_0_ found after the correction does not deviate by more then 3.3% from the number of generated tracks (Table 1).

**Table 1:** Efficiency correction.

Efficiency (%)	*N*_2_	*N*_3_	*N*_4_	*N*_3_ + *N*_4_	*N*_0_ found	Deviation (%)
100	396	72	114189	114261	114261.0	0.0
90	5697	32578	75904	108482	114110.6	0.1
70	30441	47063	27345	74408	114432.9	−0.2
50	42916	28431	7176	35607	112647.5	1.4
30	30357	8669	948	9617	110546.9	3.3

### Muography of the crater region

**Figure 6:**
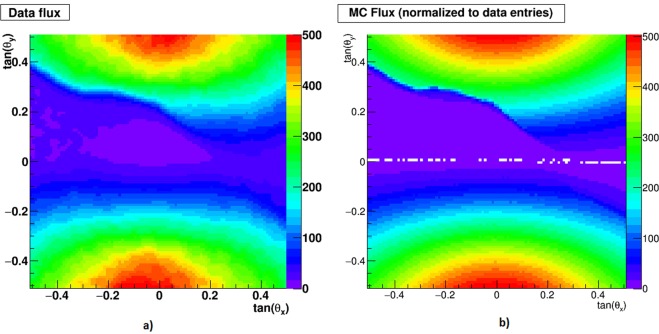
Two-dimensional histograms of the tangents of horizontal (*θ*_*x*_) and elevation (*θ*_*y*_) angles of the muon tracks, over the full angular acceptance of the detector: (**a**) data, (**b**) Monte-Carlo simulation, with total entries normalized to data. The color scale represents the number of entries in the bins.

Figure 6 shows a comparison of the distributions of the tangents of the horizontal (*θ*_*x*_) and elevation (*θ*_*y*_) between angles of the muon tracks from data (a) and from a Monte Carlo simulation (b), over the full angular acceptance of the detector. Evaluation of the expected muon flux has been performed taking as an input the muon flux parametrization^38^ with a lower cutoff energy of 1 GeV. The morphology of the volcano is described by a DEM with 10 m resolution taken shortly after the end of data-taking and provided by the Italian Civil Protection (LIDAR aerial survey performed by the Italian Department of Civil Protection in June 2012). The volcano is described as uniformly consisting of rock with a reference density of 2.2 g/cm^3^ which is in the range found by^39^ for the summit region. The detector response was studied using the Monte-Carlo simulation package FLUKA.

**Figure 7:**
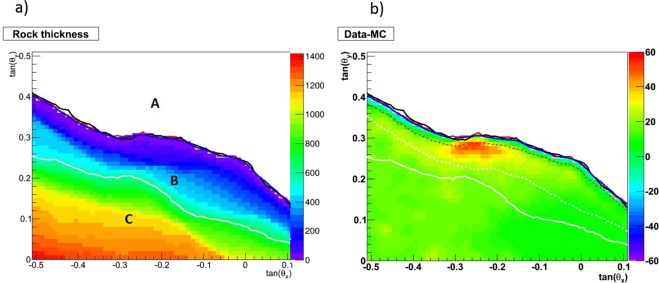
(**a**) Rock thickness and mountain profile as seen by the detector, given with 10 × 10 mrad^2^ binning. The color scale is the rock thickness in meters. The white profile gives the statistical sensitivity limit, as defined in the text. (**b**) Difference between the observed muon flux and the one expected from Monte Carlo simulation over an angular range centered at crater region. Color scale represent muons counts. The average density ranges in between 1.4 and 2.2 *g*/*cm*^3^ above the sensitivity limit.

**Figure 8:**
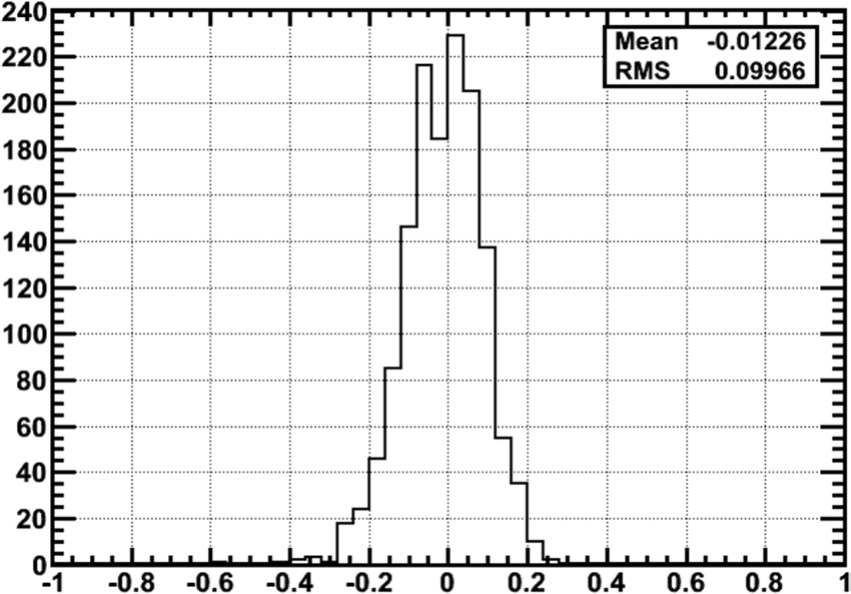
For each angular bin in the free sky region the relative spread between expectation and data is calculated.

Figure 6a and b show the same mountain profile that is given on the photo in Fig. 2a for the crater region. The highly populated zones above the mountain correspond to the muon flux from the free sky, which is maximal at the Zenith and decreases towards the horizon. To evaluate the agreement between data and simulation we plotted the relative difference calculated bin by bin in a free sky region (Fig. 8). The agreement found is within 10%, giving us an estimation of the achieved measurement accuracy. The free sky on the side opposite to the mountain is mirrored at negative angles. A band extending above and below the line of horizon extrapolated inside the mountain is dominated by background^11^ mainly attributed to a soft electromagnetic component scattered through the atmosphere or nearby rocks.

The two-dimensional histogram in Fig. 7a gives – in a color scale – the rock thickness crossed by muons in the crater region, using the DEM referred to the detector position and direction. The red contour gives the mountain profile, defined by 1 m rock thickness crossed by the muons. The black profile is derived from the measured muon flux, shown in Fig. 6a. The blue profile is derived from the MC flux, shown Fig. 6b. The angular agreement between all tree curves is good, being propagated to the distance from the detector to the target it stay within a few meters.

The white curve in Fig. 7a presents the profile corresponding to a flux of 2.3 muons (90% confidence level in case of zero background) per 20 × 20 mrad^2^ bin (about 10 × 10 m^2^ projected at the crater), according to the MC simulation. Three regions are indicated in the figure: A – free sky region, B – region accessible to muography and C – deep rock. The white profile is here considered as the sensitivity limit in the presented configuration in case of zero background. To estimate the background the data region with low theta-angle corresponding to the deep rock where the signal is expected to be at zero level was used.

Figure 7b shows the difference between observed and simulated muon flux in the crater region, normalized to the former in a region of the sky of 200 × 100 mrad just above the crater. The dashed white curve defines the lower bound of the 95% confidence level sensitivity region when also the background is accounted for. Therefore, in this study we are sensitive to the region above the dashed line. We observe a clear excess of muon flux in the crater zone (Fig. 7b), from the surface to the depth of about 50 m, which highlights the presence of lower integrated density region with respect to the one used in DEM. The density anomaly extends laterally for about 200 m below the crater region. In the anomaly region 30–40% muons excess indicates decrease of the average density along the muon path down to 1.4 g/cm^3^. The retrieved density estimation is relative and depends on the reference rock density^39^ which was taken as 2.2 g/cm^3^. There are three major components contributing to the uncertainty of this value, coming from the expected muon flux, rock thickness and the statistical error of the measured flux.

The uncertainty of the expected (simulated) flux comes mainly from fluctuation of the low-momentum part of the muon spectrum which can not be entirely rejected (Fig. 4). The spread between expected and measured values in the sky region (Fig. 8) provides the estimation of this effect(10%). Rock thickness uncertainty is defined by the DEM precision (10 m) and by the quality of angular matching between MC and data. This component becomes dominating above the red dashed line corresponding to 40 m of rock on the Fig. 7b. On the other hand, the statistical uncertainty is the leading contribution below the white dashed line. Summing up all three contributions we estimate an uncertainty for the density of 18% in the anomaly region, 10% being statistical and 15% systematic.

## Discussion

The events occurred at Stromboli in recent years have highlighted the potential impact of the crater region dynamics and the Sciara del Fuoco evolution on the risk level in the island. Actually, during an effusive phase at the end of 2002, large landslides occurred on the Sciara del Fuoco slope^40^ produced on December 30, 2002 a tsunami with waves up to 10 m high along the coasts of the island^41–43^, that caused severe damage to the coastal area. Furthermore, on April 5 2003, a paroxysmal explosion occurred in the crater region^44,45^.

In 2007, another effusive phase had a strong impact on the crater area and Sciara del Fuoco flank^46–48^. Also this eruption was accompanied by a paroxysmal explosion, on March 15th, preceded by intense fracturing of the crater region^49^. After the 2007 effusive eruption, Stromboli’s activity showed some changes. The frequency of occurrence of “major explosions” and effusive phases due to overflows from the summit craters increased. The changes of the eruptive activity occurred at Stromboli from April 2007 to December 2012 is interpreted as the effect of “a wider and hotter uppermost conduit, initiated by movements that occurred in the Sciara del Fuoco after the 2002 landslide events^50,51^ that followed the graben-like collapses that occurred during the 2007 eruption, which involved the entire summit crater zone”^52^.

This interpretation suggests a strong control of the crater area structure and the Sciara del Fuoco slope on the volcanic dynamics of Stromboli Island. Studies, based on electrical resistivity and self-potential measurements^53,54^, on gravity data^39^ aeromagnetic surveys^55^ and on seismic tomography^56^ have been carried out to gain insights into the structure of Stromboli edifice. In particular^53^, conducted combined measurements of electrical resistivity, self-potential, CO_2_ and temperature to study the shallow hydrothermal system of the Stromboli volcano. They identify a conductive body in the summit part of the volcano (crater zone) that they interpret as the main hydrothermal system of Stromboli.

Seismological studies, focused on seismic source mechanisms, have shown that the shallow magma transport system is characterized by a crack with NE-SW direction dipping about 60° to the North-West and spanning up to the crater region^57^. Therefore, our muography experiment is focused on the crater region.

Before the beginning of the muography (started on October 22, 2011), on August 2, 2011 an effusive phase occurred due to lava overflow from NE crater^58^, accompanied by remarkable explosions. Two major explosions occurred on September 5 and September 9. During the first month of data-taking the eruptive activity decreased and returned to the normal level. Two major explosions occurred on February 15 and March 6, 2012, however they did not cause significant changes of the crater region morphology. The experiment ended on March 24, 2012.

Our experiment allowed us to obtain the first muon absorption radiography of the crater region of Stromboli. The muon track reconstruction provided an image of the crater area of Stromboli volcano, with a resolution of about 10 meters in the center of the target area. The muography, shown in Fig. 7b, highlights a low-density zone in proximity of the NE crater area. The elongation of the low-density zone towards SW (right on the Figure) indicates that the density anomaly also includes the crater valley at the summit of the volcano, where other eruptive vents are located. The anomaly of the flux along the muon path through the crater zone indicates a density decrease down to 1.4 g/cm^3^. The muon flux anomaly with dimensions of about 200 × 50 m corresponds to the graben-like region of^52^ shown in Fig. 1. Our findings are consistent with the above geophysical surveys^39,53,55^ indicating that low-density, high-porosity, pyroclastic deposits characterize the collapsed zone of the crater area. Actually^55^, found a magnetization low in the crater zone. This demagnetization is interpreted as the consequence of the heat of conduits and/or hydrothermal activity in addition to the thick accumulation of low magnetic pyroclastic rocks. This result is consistent with the gravimetric survey performed by^39^, who found a low-density anomaly (2100–2250 kg/m^3^) on the Pizzo ridge, associated to low-density, high-porosity and high-permeability pyroclastic and scoria deposits. In particular, the sector of the volcano imaged by our muography experiment includes, along the muon paths, part of the main hydrothermal system highlighted by^53^ and also the south-west sector of the summit zone which is characterized by high resistivity. Basically, from our exposure site we find a low density anomaly that is consistent with the presence of low density pyroclastic material and degassing conduits that can supply heat to the hydrothermal system.

Therefore the muography allows us to image the collapse structure that was created by the 2003 and 2007 eruptions, which controls the eruptive style of Stromboli after 2007.

The chosen value of the reference rock density (2.2 g/cm^3^) leads to a density of about 1.4 g/cm^3^ in the crater graben anomaly area. The density anomaly can be well explained by the presence of porous incoherent material that fills the collapse structure in the summit crater zone. A low density comparable with the anomaly of Stromboli has been reported by^59^ at La Soufriere volcano.

Precise muography measurements of deep rock regions requires the ability for cutting off the low-momentum particles presenting the main source of the background as shown in^60^ and^61^. The use of an Emulsion Cloud Chamber (ECC) detector installed in the same position with sufficient number of sensitive layers interleaved with high-density absorbers will extend the investigation reported in this paper to the region of about 100 m under the crater.

## Methods

The nuclear emulsion is in principle similar to the photographic emulsion, but it is sensitive to single ionizing particles. AgBr crystals interspersed in a gel matrix are sensitized by ionization caused by charged particles, producing a latent image. After the chemical process of emulsion development, grains of metallic Ag grow after nucleation by the latent image sites, normally up to the size of 0.5–1 *μ*m. The path of a ionizing particle is thus marked by a sequence of grains. The average number of grains building up the track in the sensitive emulsion layer depends on the strength of ionization^62^.

In the emulsion used at Stromboli, the typical linear density is 25 grains/100 *μ*m. Latent images of particles tracks are continuously stored in emulsion during the timespan of data taking, until they are made visible following their chemical development as for photographic emulsion.

The nuclear emulsion technique came to life in the years 1946–1947 with the Nobel Prize discovery of the short-lived *π* meson (*pion*) in an exposure at high altitude to particles generated in cosmic-ray interactions in the atmosphere^63,64^. A true re-birth nuclear emulsion technique occurred in 1990 by the with the development of emulsion analysis by automated microscopes^65,66^ that opened the way to large scale applications. Profiting of the developments realized in the OPERA experiment, the nuclear emulsion technique became readily applicable to muography and was used for the pioneering observations at the Mt. Asama volcano^2^. Recent developments of even faster scanning systems operating in a wide angular range^35,67–69^ further extends the applications area for this technique.

Thanks to the sub-micrometric resolution, the nuclear emulsion has since the beginnings proven to be a privileged tool for observing the decays of short-lived particles, as well as a precision particle tracker in very compact detectors for applications, such as muography. Emulsion detectors do not require electricity supply nor maintenance. The latter features were considered as a must in the choice of the detector technique for harsh environment characterizing the slopes of the Stromboli volcano.

The basic detector element is an emulsion film that is technically sophisticated but mechanically simple. The OPERA films^32^ have 12.5 × 10.2 cm^2^ size and consists of 44 *μ*m thick emulsion layers deposited on either side of a 205 *μ*m thick plastic base. Micro-tracks are reconstructed from aligned grains in each single emulsion layer (Fig. 5). Aligned micro-tracks are connected across the plastic base to form a so-called *base - track*. This is characterized by a higher precision thanks to the longer lever arm given by the thickness of the plastic base and to the well-defined geometry in spite of the emulsion distortion in drying-out and shrinking after the chemical development.

Emulsion films are piled-up on stacks to provide several measurements points for the reconstruction of through going particle tracks. Typically, intrinsic angular resolutions are of the order of a few mrad. The emulsion stack used at Stromboli consists of four emulsion films and includes a 5 mm thick plate of passive material (stainless steel). The latter improves the rejection of background tracks from low-energy stray particles, either by absorption or by enhancing misalignments of track segments.

Inserting plates of passive material provides information on particle properties through the observation of their interactions. The most striking example is the ECC-based detectors^70^, where alternating emulsion films and plates of passive material integrates in a single very compact structure with high precision tracking and capabilities of particle identification and momentum/energy measurement. This technique has been used in OPERA^33^, where the passive material consists of 1 mm thick lead plates. The insertion of passive material is an effective tool for background reduction in muography.

Our detector, with total active area of 0.96 m^2^, consisted of eight modules assembled side-by-side on an common support frame. The overall dimension were about 0.7 × 3.5 m^2^ and the total weight was 250 kg (Fig. 5). In each module of 0.12 m^2^ active surface, ten emulsion stacks were assembled side-by-side. The emulsion stacks were tightly enclosed inside robust hermetic boxes made of aluminum and stainless steel, allowing for on-site emulsion mounting and replacement. The assembly scheme in Fig. 5 shows the emulsion stacks with two emulsion films sticked on each side of the stainless steel plate, the frame and the covers of the box, as well as rubber sheets between emulsion films and box covers.

Muon tracks are obtained in a two-stage process. In the first step (emulsion readout), image data stored in the films are read out and converted into digital representations of micro-tracks in each emulsion layer. In the second-step (offline analysis), these are used to perform a full volume reconstruction of muon tracks crossing the detector. They follow the methodologies developed for OPERA.

The emulsion readout consists in “scanning” each single emulsion layer in the three spatial dimensions, transforming visual into digital data and performing a first phase of data processing that gives micro-tracks as output. The emulsions from the Stromboli exposure were scanned by means of high-speed automated microscopes developed in Europe for OPERA - clones of the so-called European Scanning System (ESS) - that operate at a nominal speed of 20 cm^2^/hour^71,72^. An evolved system based on the same hardware^35^ was used to analyze about a half of the data sample.

The objective of the microscope has working distance of a few hundred micrometers and a depth of field of a few *μ*m. It is carried on a vertically sliding optical tube with the camera sensor at its end. By moving the optical tube, the focal plane is changed, thus allowing for a 3D optical tomography of 15 consecutive images and a micro-tracks recognition as sequences of aligned grains. At least 8 grains are required^73^.

Emulsion films are kept fixed to a film-holder carried by a microscope stage capable of motion on two orthogonal axes (X-Y). By moving the stage with steps corresponding to the field of view size (260 × 300 *μ*m^2^) the full emulsion surface readout (scanning) is performed.

The offline track reconstruction procedure starts by forming the base-tracks finding pairs of aligned micro-tracks across the plastic base. Base tracks further improve the rejection of fakes and, as already pointed out, improve the angular resolution that attains a few mrad^73^.

The reconstruction of full tracks requires connecting base-tracks in the sequence of emulsion films. The crucial point is the film-to-film alignment, with a micrometric accuracy necessary to separate muons accumulated during the exposure from fake associations (nuclear emulsions have no trigger and are sensitive since production until development, hence piling up the tracks of all ionizing particles passed through). The alignment is performed in turn for all pairs of consecutive films by applying a pattern recognition technique to the arrays of base-tracks. The first order approximation model suitable at the cm scale is an affine deformation. Emulsion films are aligned with the accuracy of a few *μ*m^73^.

After application to the base-tracks of the affine transformation parameters found for each film, reconstruction of passing-through long tracks becomes possible. For Stromboli data this was done by the Kalman-filtering technique described in^74^. In case of multilayer detector this procedure has great rejection potential against fake and low-momentum tracks considered as a background for the muography exposure.

## Data Availability

The datasets generated and analyzed during the current study are available from the corresponding author on reasonable request.
